# Successful pregnancy in a chronically hemodialyzed patient with end-stage renal failure

**DOI:** 10.4103/0971-4065.50678

**Published:** 2009-01

**Authors:** G. Orlowska-Kowalik, T. Malecka-Massalska, A. Ksiazek

**Affiliations:** Nephrology Department of Medical University, Jaczewskiego Street 8, 20-950 Lublin, Poland

**Keywords:** End-stage renal failure, hemodialysis, hypertension, pregnancy

## Abstract

A 36 year-old female with chronic kidney failure due to hypertension and who was being treated with hemodialysis for eight months, was admitted to the hospital on the suspicion of being pregnant. Gynecological examination and ultrasound scan confirmed the pregnancy. Gestation was diagnosed in the 29^th^ week after the patient felt fetal movements. Intensification of the dialysis treatment was started immediately after the diagnosis was made.

## Introduction

In women with chronic kidney disease pregnancy may prompt the initiation of dialysis. Pregnancies in patients with end-stage renal failure present a great risk for the mother - volume overload, exacerbation of hypertension, eclampsia, and for the infant - respiratory distress syndrome, leukopenia, thrombocytopenia, reduced birth weight, adrenocortical insufficiency, infection.

The effects of improved dialysis patients ability to conceive could increase likelihood of conception and thus the reported incidence of pregnancy. We report the case of pregnancy in women who was chronically hemodialysed.

## Case Report

A 36 year-old female patient was admitted to the Nephrology Department with dyspnea, hypertension, edema of the lower extremities, and ascites. The results of laboratory findings showed serum levels of urea to be 318 mg/ dL; creatinine 14.7 mg/dL; pH of 7.2; PTH 5.71 pg/mL; Hb 8.01 g/dL, Ht 27.7%; RBC 2,720,000 cells/μL; WBC 5.280 cells/μL; PLT 203,000 cells/μL; Na 137 mEq/L; K 4.6 mEq/L; ionised calcium 0.47 mmol/L. Urine analysis showed an albumin level of 100 mg/dL. Chest X-ray examination demonstrated symptoms of stasis in the pulmonary circulation, similar to pulmonary edema. Ultrasonography showed a small left kidney with a narrow renal cortex of 62 × 32 mm and an RR of 150/100 mmHg. The patient passed less than 2,000 mL of urine daily. Six years ago, the patient had undergone a right-side nephrectomy due to nephrosclerosis of this kidney and hypertension. The creatinine level had been 2 mg/dL following the operation. After that, the patient did not go for any check-ups and was not treated. In her medical history, the patient had two spontaneous deliveries 13 and 11 years ago. The last menstruation was four months before hospital admission. Due to advanced renal failure, renal replacement therapy was started with hemodialysis. The patient's anemia made her a subject for erythropoietin treatment. Within two weeks of dialysis, it was possible to reduce the patient's weight by 18 kg. The patient was dialyzed regularly, three times a week for four hours. After eight months of dialysis, the patient informed the nephrologist that she might be pregnant. Gynecological and ultrasonograph examination confirmed the presence of a live fetus at 29 weeks of gestation. From then onwards, the dialysis therapy was intensified to reach six procedures per week, each lasting for 3.5 hours. The results of laboratory findings showed: Hb of 8.2 g/dL, Ht 22.7%, RBC 2,540,000 cells/μL, WBC 12,510 cells/μL, PLT 196,000 cells/μL, urea 34.6 mg/dL, creatinine 4.79 mg/dL, CRP 1.8 mg/L, ASPAT 34 U/L, ALAT 20 U/L, Na 138 mEq/L, K 5.1 mEq/L, Ca 8.85 mg/dL, total protein 6.23 g/dL, albumin 59.5%, and RR of 120/80 mmHg. The erythropoietin dose was increased to 12,000 U per week which made it possible to maintain hemoglobin levels within 10–11 g/dL. The patient took folic acid, vitamin B6, and Alphacalcidol 0.25 ug three times a week, as well as calcium carbonate, iron, and salicylic acid. The patient was dialyzed by using bicarbonate dialysate (without heparin) and GFS 16 dialyzators that were not reutilized. Blood flow through the dialyzator ranged from 280 to 300 mL/min and urea concentrations before dialysis hovered around 50–70 mg/dL. Interdialytic weight gains even reached 2.5 kg. The patient passed around 100–150 mL of urine daily. Ultrafiltrations were performed in such a way as to avoid pressure falls during the procedure. Dry weight gain amounted to about 500 gm every ten days. The patient was informed about the proper diet. The calories' intake was 35 kcal per kg of body weight and the protein's intake was 1.5 g per kg of body weight. From the moment of gestational diagnosis, fetal development and condition were monitored based on ultrasonography examination. The first examination revealed hydramnios and features of intrauterine growth retardation, but no fetal developmental anomalies could be observed. Fetal weight gain and persistent hydramnios were observed in the subsequent examinations. By the end of 32 weeks of gestation, the patient started to suffer from a rather strong itch; ALAT was found to have increased to 47 U/L. At 34 weeks of gestation, cholestasis features were found to have intensified and hydramnios and cervical dilatation at 1 cm were observed with the pelvic lie of the fetus; hence, the patient was qualified for cesarean section delivery. Epidural anesthesia at the L4-L5 interspace was performed with the use of 2.2 mL Marcaine 0.5% (Bupivacaine). Intravenous epinerphine, 15 and 25 mg, was used intravenously in the beginning and at a later stage of the cesarean section procedure respectively. Five units of Oxytocin were used intravenously. Neither surgical nor any other kind of problems were observed. A male baby with a birth weight of 1670 g was born. The Apgar score in the first minute after birth was 6; it reached 8 points in the third minute after birth. The newborn was diagnosed with signs of intrauterine growth restriction, general edema, and respiratory failure with tissue perfusion disorders, requiring 48 h intubation of the newborn. Oliguria was also observed in the first 24 hours and the baby was discharged after 46 days. After the delivery, the mother returned to the system of three dialyses per week.

## Discussion

The incidence of pregnancy in women on dialysis is low.[1,2] Impaired ovulation, amenorrhea, endocrinal abnormalities, anemia, and reduced libido are common in dialysed women.[2,3] Pregnancies in patients with end-stage renal failure present a great risk for the mother: volume overload, exacerbation of hypertension, eclampsia, and for the infant: respiratory distress syndrome, leukopenia, thrombocytopenia, reduced birth weight, adrenocortical insufficiency, and infection. Only half of the infants born to women on chronic dialysis survive.[1–6]

Diagnosing gestation among women undergoing dialysis is very difficult. The initial symptoms of pregnancy-menstruation disorders, nausea, or vomiting are usually associated with advanced renal failure and dialysis complications. Therefore, gestation diagnosis usually takes place after the 16^th^ week.[1] In our case, gestation was diagnosed in the 29^th^ week after the patient felt fetal movements.

Treating pregnant women with hemodialysis raises numerous problems and requires a modification of the routine procedure. These modifications are aimed, on the one hand, to maximally reduce the influence of uremic toxemia on fetal development, and on the other hand, to avoid the complications of dialysis which could provoke a premature interruption of gestation. The dialysis dose should be increased so that predialysis BUN concentrations are maintained below 50–60 mg/dL.[1,3,5,7–10] The preferred methods are procedures that can be performed more frequently (6–7 times a week) rather than less frequent ones that are longer. In our patient, the dialysis dose was increased to six sessions a week after gestational diagnosis (21 hours per week). It is possible that the patient's high interdialytic weight gains before the gestational diagnosis, contributed to the development of hydramnios. Intensive dialyzing requires a modification of the composition of the dialysis liquid. Insufficient intake of potassium salt with an increase in the dialysis dose can lead to a potassium deficit. Therefore, the concentration of K^+^ in the dialysis liquid should be increased to 3.0–3.5 mmol/L, and its concentration in the patient's blood serum should be monitored and controlled several times a week. Despite an increased demand for calcium during gestation, its concentration in the dialysis liquid should be 1.25 mmol/L. Administering dialysis liquid with a calcium concentration of 1.75 mmol/L equals about 900 mg intake of calcium during each procedure. Proper skeleton formation requires a total of about 25–30 g of calcium. In our patient, frequent controls pointed to the low level of ionised calcium. Therefore, we administered dialysis liquid containing Alphacalcidol and a calcium concentration of 1.75 mmol/L during dialysis. Particular attention should be given to controlling the phosphate concentration in the mother's blood, as hyperphosphatemia negatively affects skeleton development. Due to increased progesterone levels during gestation, hyperventilation is observed which causes respiratory alkalosis. Frequent dialyses also provide excess bicarbonate buffer which may incur the risk of alkalosis development. Therefore, it is recommended that bicarbonate concentration in the dialysis liquid is reduced to 25 mmol/L.[1,3,10,11] The change in a pregnant woman's dry weight is of utmost importance. Excessive ultrafiltration during dialysis leads to hypovolemia and blood pressure fall that are dangerous for the fetus. Overhydration, in turn, incurs the risk of hypertension, which can be equally threatening for the fetus. Retrospective analysis of the course of gestation in dialyzed pregnant women indicates that there is usually a slight weight gain of 1.0–1.5 kg in the first trimester. In the second and third trimesters, weight gain is linear and amounts to about 500 g every ten days.[10] However, it is necessary to carefully monitor fetal development and the amount of amniotic fluid by means of ultrasonography. In our patient, hydromnia was diagnosed along with gestation. Anemia usually worsens in dialyzed women after conception and the demand for erythropoietin and iron increases. If transferrin saturation is below 30%, intravenous iron supply is recommended at the onset of gestation. Erythropoietin does not cause any significant side effects in the fetus. Due to serious anemia in our case, the erythropoietin dose was increased to 12,000 U per week.

There are no current contraindications to administering heparin. Heparin does not cross the placental barrier and is not teratogenic.[11] However, it is recommended to administer minimal doses of aspirin to prevent preeclampsia.[10,12] Hypertension is the most frequent complication in 42–80% of pregnant patients. It is the reason for postpartum hemorrhage, detachment of the placenta, and anemia in at least half of all cases.[5,13] Our patient suffered from hypertension and required hypertension treatment even before the start of hemodialysis treatment and at the onset of renal replacement therapy. After getting down to the proper dry weight, her blood pressure normalized and remained normal throughout the course of the gestation. Due to the risk of developmental anomalies of the fetus incurred by the contact with formaldehyde or ethylene oxide, one-time use of biocompatible dialyzators is recommended. In the study by Bagon *et al*., 66% of pregnancies were ended by cesarean section.[9] This method is a necessity in the face of frequent gestational complications. It is most frequently performed due to the premature rupture of fetal membranes. It is assumed that cesarean section should be performed only for obstetric indications and not due to renal failure. The duration of end-stage renal failure appears to have an important influence on the likehood of pregnancy as well as the pregnancy outcome.
